# An Efficient Protocol for Plantlet Regeneration via Direct Organogenesis by Using Nodal Segments from Embryo-Cultured Seedlings of *Cinnamomum camphora* L.

**DOI:** 10.1371/journal.pone.0127215

**Published:** 2015-05-11

**Authors:** Li Du, Yongpeng Li, Yao Yao, Liwei Zhang

**Affiliations:** School of Life Science and Technology, Nanyang Normal University, Nanyang City, Henan Province, P. R. China; INRA, FRANCE

## Abstract

A simple and efficient plantlet regeneration protocol *via* direct organogenesis was established for camphor tree (*Cinnamomum camphora* L.). Stem segments with one node (SN explants) from embryo-cultured seedlings (EC seedlings) were used as explants. Murashige and Skoog (MS) medium supplemented with 0.5 mg/L 2, 4-dichlorophenoxyacetic acid and 2.0 mg/L 6-benzyladenine was used to induce cotyledonary embryo germination. This medium was also used for EC seedlings propagation and adventitious bud induction from SN explants. Regenerated plantlets were cultured on hormone-free MS medium for elongation and root induction. The regeneration capability of SN explants was compared by using EC seedling lines established in this research. EC seedling line EL6 exhibited the highest adventitious bud induction frequency (91.7%) and the highest number of buds per responding explant (5.2), which was considered as the most efficient EC seedling line for further gene transformation research.

## Introduction

*Cinnamomum camphor* (commonly known as camphor tree or camphor laurel) is a large, handsome evergreen tree that grows up to 20~30 m (66~98 ft) tall. Whole plant is strongly camphor-scented. In spring, it produces masses of small fragrant green-white or yellowish flowers. Camphor tree is native to China south of the Yangtze River, Taiwan, southern Japan, Korea and Vietnam and widely cultivated in warm regions of the world as an ornamental street or shade tree [1–6]. The introduction of camphor tree to landscape gardens of Northern China is considered to enrich local evergreen tree species and improve garden landscapes in winter. However, the poor tolerance to low temperatures is a major limiting factor for camphor tree to its successful introduction into Northern China, which has long and cold winter.

Gene transfer technologies offer an approach for the transfer of genetic traits, such as cold tolerance, into germplasms. For this to be viable, a reliable and efficient plantlet regeneration system need to be established firstly [7–9]. For camphor tree, the currently available regeneration protocols were based on somatic embryogenesis from cotyledonary embryo explants. However, these protocols were limited by low regeneration frequencies and long regeneration periods [2, 3, 5, 6]. In the study of transgenic camphor tree, various antibiotics as selective agents in culture media were reported to have a strongly negative effect on plantlet regeneration from transgenic embryogenic calli [2, 5]. To the best of our knowledge, there have been few researches reported on transgenic regenerated plantlet *via* somatic embryogenesis of camphor tree. Therefore, these issues above might make somatic embryogenesis regeneration system impossible to provide efficient acceptors for camphor trees transformation [2].

Direct and indirect shoot organogenesis from various explants, including cotyledonary nodes [9], hypocotyls [10], leaf [7, 11, 12], mature internodal stem segments [8, 13] and stems with nodal segments [13–16], have been reported as effective explants for many plant species. Direct organogenesis regeneration from explants, omitting the callus induction phase, was desirable especially in modern breeding where increasing rapidity and reducing costs of regeneration were essential [12, 17]. Among woody plants, plantlet regeneration *via* organogenesis has been achieved easily for juvenile explants from seeds or seedlings [18–21]. However, seeds from cross-pollinated species were not suitable explants supplier for mixed genetic background. Additionally, plantlet regeneration from nodal stems or shoots apices explants were thought to be a result of proliferation of pre-existing meristems, making these systems inefficient for transformation studies [22]. Therefore, the selection of juvenile explants supplier with same genetic background and development of an efficient protocol *via* direct organogenesis have the potential to contribute to a more stable genetic transformation for camphor tree.

Extensive work has been carried out for juvenile explants supplier selection and direct organogenesis induction of camphor tree, including embryos germination induction, screening and propagation of highly proliferation embryo-cultured (EC) seedling lines, adventitious buds induction from explants, elongation and rooting of regenerated shoots. To eliminate the influence of pre-existing meristems, stem segments with one node removed axillary buds (SN explants) were prepared for adventitious bud induction. In this research, an efficient plantlet regeneration system in camphor tree *via* direct organogenesis was established through nodal segments from embryo-cultured seedlings, which could be used in future transformation studies.

## Materials and Methods

### Media and culture conditions

S1 medium was used for several purposes in this research, which comprised Murashige and Skoog (MS) basal medium with 30 g/L sucrose, 8.0 g/L agar, 0.5 mg/L 2, 4-dichlorophenoxyacetic acid (2, 4-D) and 2.0 mg/L 6-benzyla-denine (BA). This medium could be used for embryo germination induction, propagation of EC seedlings and adventitious bud induction from SN explants of EC seedlings. Hormone-free MS medium containing 30 g/L sucrose and 8.0 g/L agar was used as elongation and root induction medium for regenerated plantlets. The pH of all media was adjusted to 6.0 prior to autoclaving (121 C, 20 min). Cultures were incubated at 24 ± 2°C with a daily 16-h illumination regimen of 40 μmol m^-2^ s^-1^ photosynthetically active radiations provided by white fluorescent lights except cotyledonary embryo germination culture in the dark.

### Plant materials for EC seedlings

Adult camphor tree blooms between April and May in Nanyang, Henan province of China, and the fruits begin to swell during June and July. The fruit matures in early November. In this research, immature camphor tree fruits were collected from one selected excellent donor tree on the campus of Nanyang Normal University at the end of July 2013. All the immature fruits picked were of the same size. The fruit was8~10 mm in diameter and the size of cotyledonary embryo was about 2~3 mm, only one-eighth of the whole fruit. And two fleshy cotyledons of embryo appeared creamy white. The fruits were surface-sterilized by immersion in 75% (v/v) ethanol for 30 s, followed by immersion in 0.1% (w/v) HgCl_2_ for 8 min and then rinsed three times with sterile distilled water. The cotyledonary embryos were aseptically excised from the immature fruits and placed on S1 medium.

### EC seedlings propagation of *C*. *camphora*

Complete embryos were incubated on *petri* dishes containing S1 medium in the dark. After 4 weeks of culture, individual seedling was separated from black and brown cotyledons and transferred to culture vessels with the same medium. 2 weeks of light culture later, multiple shoots could be observed at the base of some single cultured EC seedlings; those seedlings were selected, designated and subcultured several times to establish EC seedling lines, whose proliferation coefficient (number of induced shoots per seedling) was over 3.0. EC seedlings rapid propagation system could be established in this way and used as an explants supplier for further direct organogenesis induction.

### Plantlet regeneration from nodal segments of EC seedlings

In order to determine the optimum components for adventitious bud induction, SN explants from the complicated genetic background (mixed EC seedling lines) were incubated on various induction media. MS medium containing 30 g/L sucrose, 8.0 g/L agar, BA (1.0, 2.0, or 3.0 mg/L) and CH (CH: Casein hydrolysate) (0 or 1000 mg/L) and supplemented with different auxins (2, 4-D or IBA) (IBA: Indole-3-butyric acid) at 0.1 or 0.5 mg/L were used as induction media. MS medium without hormones and supplements was served as a control.

The regeneration capability of SN explants was compared for five highly proliferation EC seedling lines (EL1, EL2, EL3, EL4 and EL6). The optimal adventitious bud induction medium was determined to be S1 medium from previous induction experiments; and all the explants from different EC seedling lines (different explants donor genotype)were cultured on S1 medium for regeneration comparison.

After 2 weeks of regeneration induction, the adventitious buds induction frequency (%) and the number of buds per responding explant was recorded. Each experiment was repeated twice with 30 explants per treatment.

The SN explants from EL6 were cultured on different induction media, after 2 weeks of culture, adventitious shoots derived from different induction media (Treatment S1, S5 and S6) and then were transferred to hormone-free MS medium for rootability comparison. At least 30 regenerated shoots were cultured per treatment and each experiment was repeated twice. After 4 weeks of culture, the root induction frequency (%) and the multiple roots induction frequency (%) was recorded. Shoot forming at least 3.0 adventitious roots was considered as a responding explant and scored for multiple roots induction frequency.

The plantlet regeneration system could be established by use of SN explants from EC seedlings as explants, and the explant preparation was provided in the following.

### Explant preparation

**Fig 1:**
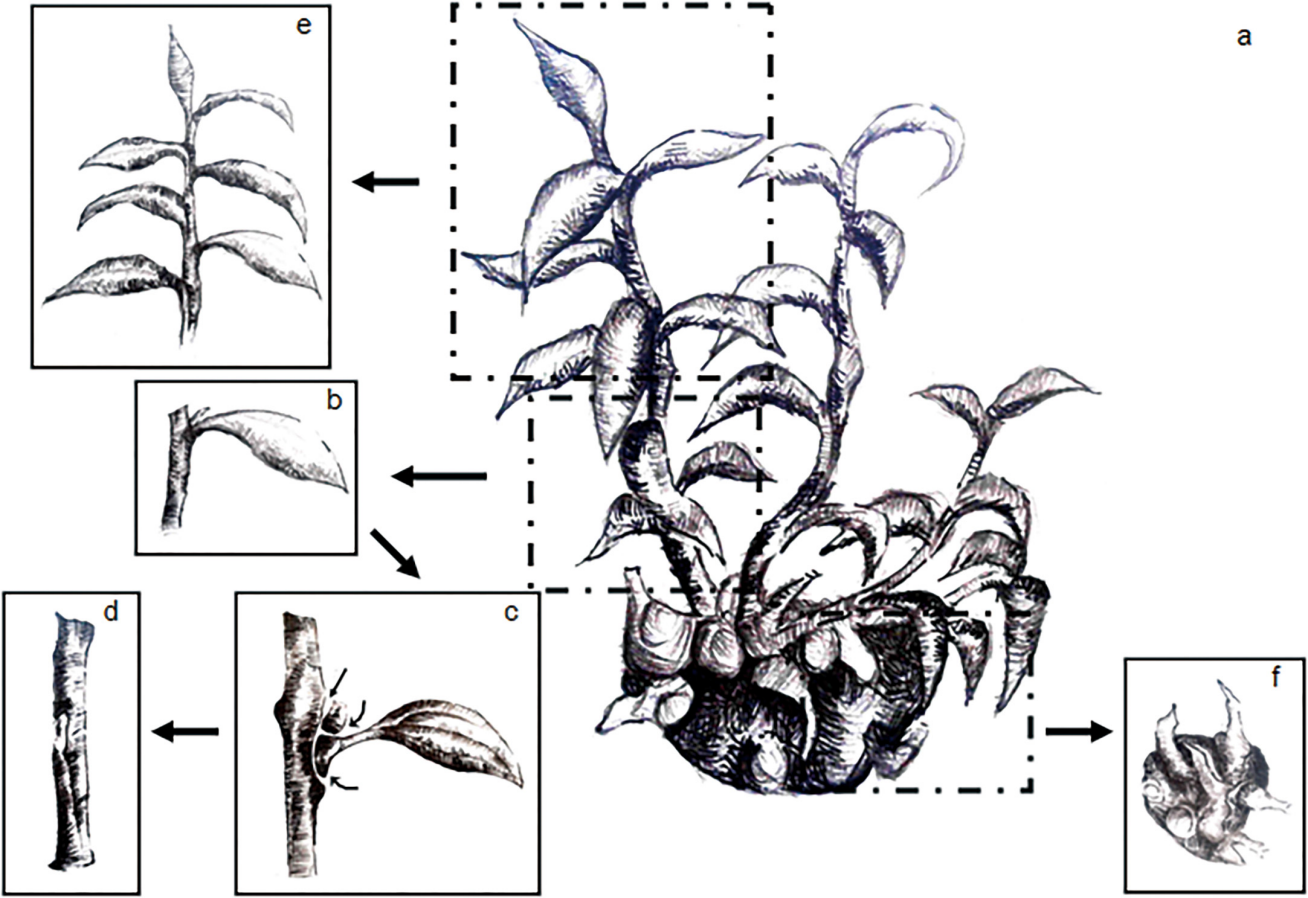
Schematic representation of the procedure adopted for preparing the explants. a) The multiple shoots induced from EC seedling; b) the stem segment removed from shoot cluster; c) the removal of axillary bud and leaflet from the stem (arrows indicate surgical excision points); d) a stem segment with one node (SN) ready for adventitious buds induction; e) a shoot with terminal bud explant (TS) ready for elongation and root induction; f) the basal section of the multiple shoots (BMS) ready for propagation of EC seedlings of *C*. *camphora*.

Different culture results could be achieved by use of different parts of EC seedlings. EC seedling with multiple shoots (Fig 1A) was obtained by use of single seedling culture. Stem segment (5~7 mm) with one node (Fig 1B)was separated from the shoot. Then the stem segment was carefully removed axillary bud and leaflet (Fig 1C). This resulted in one stem segment with one node explant (SN explants) (Fig 1D) that was then cultured in S1 medium for adventitious bud induction. The terminal shoots (TS explants; 15~20 mm) could be induced adventitious roots while being cultured in hormone-free MS medium (Fig 1E). The base of EC seedlings were cut into small portions (BMS explants), and the BMS explants could be cultured for EC seedlings propagation (Fig 1F). This procedure could also be adopted for preparing the explants for propagation and root induction of regenerated plantlet from SN explants.

### Statistical analysis

The data collected from the experiments were subjected to one way ANOVA (analysis of variance) using SPSS 17.0 software with Duncan’s multiple range test at level of significance P = 0.05. Percentage data were transformed *via* arcsine before analysis.

## Results and Discussion

### EC seedlings rapid propagation system

**Fig 2:**
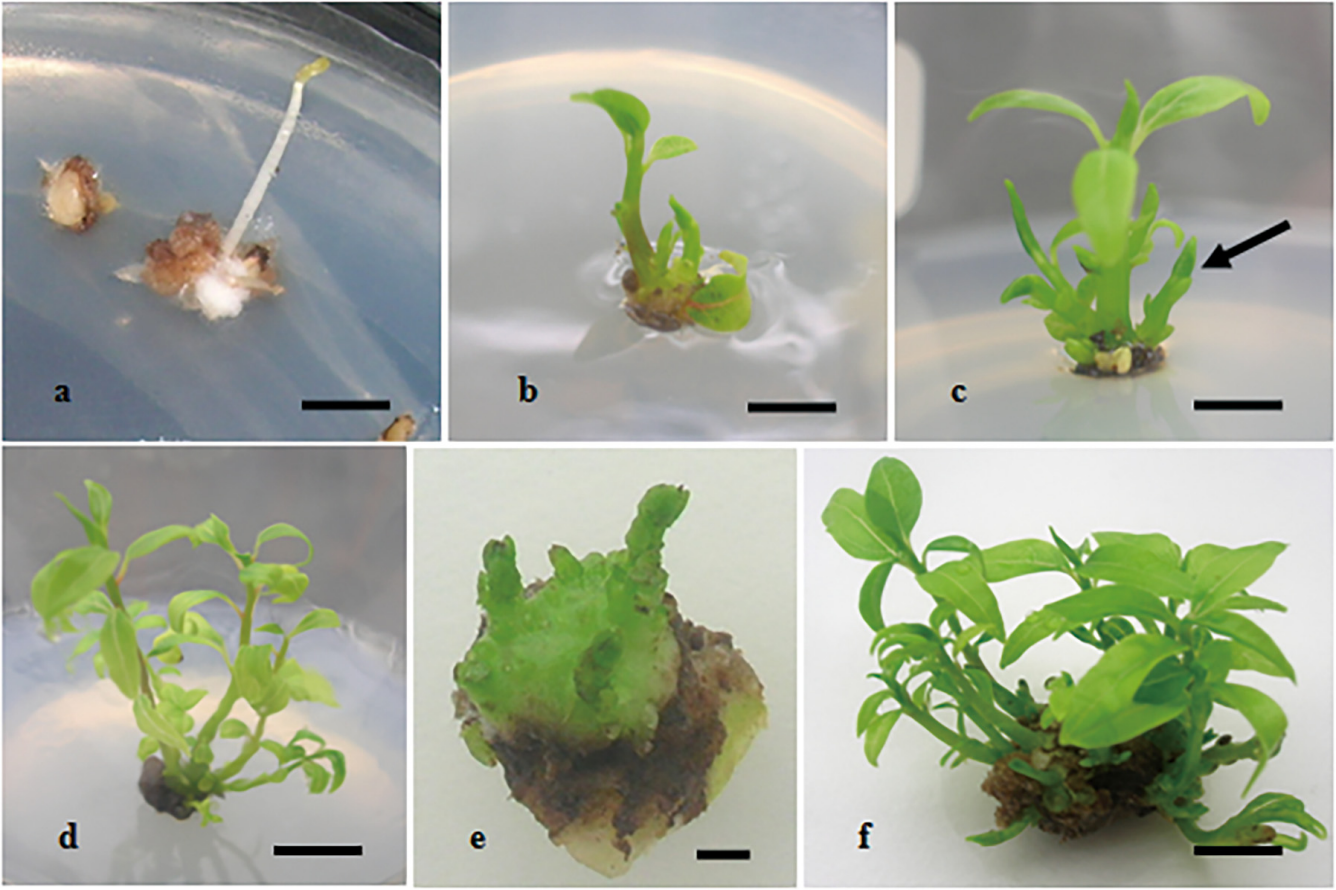
Cotyledonary embryo-cultured seedlings propagation of *C*. *camphora*. a) Germination induction for cotyledonary embryo of *C*. *camphora*, bar: 1.0 cm; b) EC seedlings transferred to the light culture, bar: 1.0 cm; c) multiple shoots sprouting from the basal section of seedling, bar: 1.0 cm; d) EC seedling line with high proliferation, bar: 1.0 cm; e) the basal section of the multiple shoots (BMS explant) from EC seedling, bar: 0.3 cm; f) the shoots proliferation of BMS explant after 2 weeks culture, bar: 1.0 cm.

Cotyledonary embryo germination was observed during the first week of culture, with tender and pale yellow germ spouted directly from embryo (Fig 2A). After 4 weeks of embryo germination induction, individual EC seedling was isolated from the dark and brown cotyledons, and was transferred to culture vessel containing S1 medium, then exposed to light. These shoots turned green and strong in the light (Fig 2B). The apical dominance of single cultured EC seedlings was not significant, with multiple shoots derived from the base of seedlings (Fig 2C). 2 weeks later in light culture, 17 EC seedlings with multiple shoots were obtained in this research. Through screening tests (proliferation coefficient, number of induced shoots per seedling below 3.0), EC seedlings with low proliferation capability were screened out; High-propagation EC seedlings were selected and propagated. Through several rounds of subculture, five EC seedling lines were formed and designated EL1, EL2, EL3, EL4, and EL6 (Fig 2D). EC seedling subculture and propagation was efficient by the use of BMS explants (Fig 2E), with multiple shoots observed (Fig 2F). The EC seedlings rapid propagation system of camphor tree were established by means of embryo culture and BMS explants subculture, and could be severed as a stable juvenile explants supplier for further direct organogenesis induction.

### Plantlet regeneration system from SN explants

**Fig 3:**
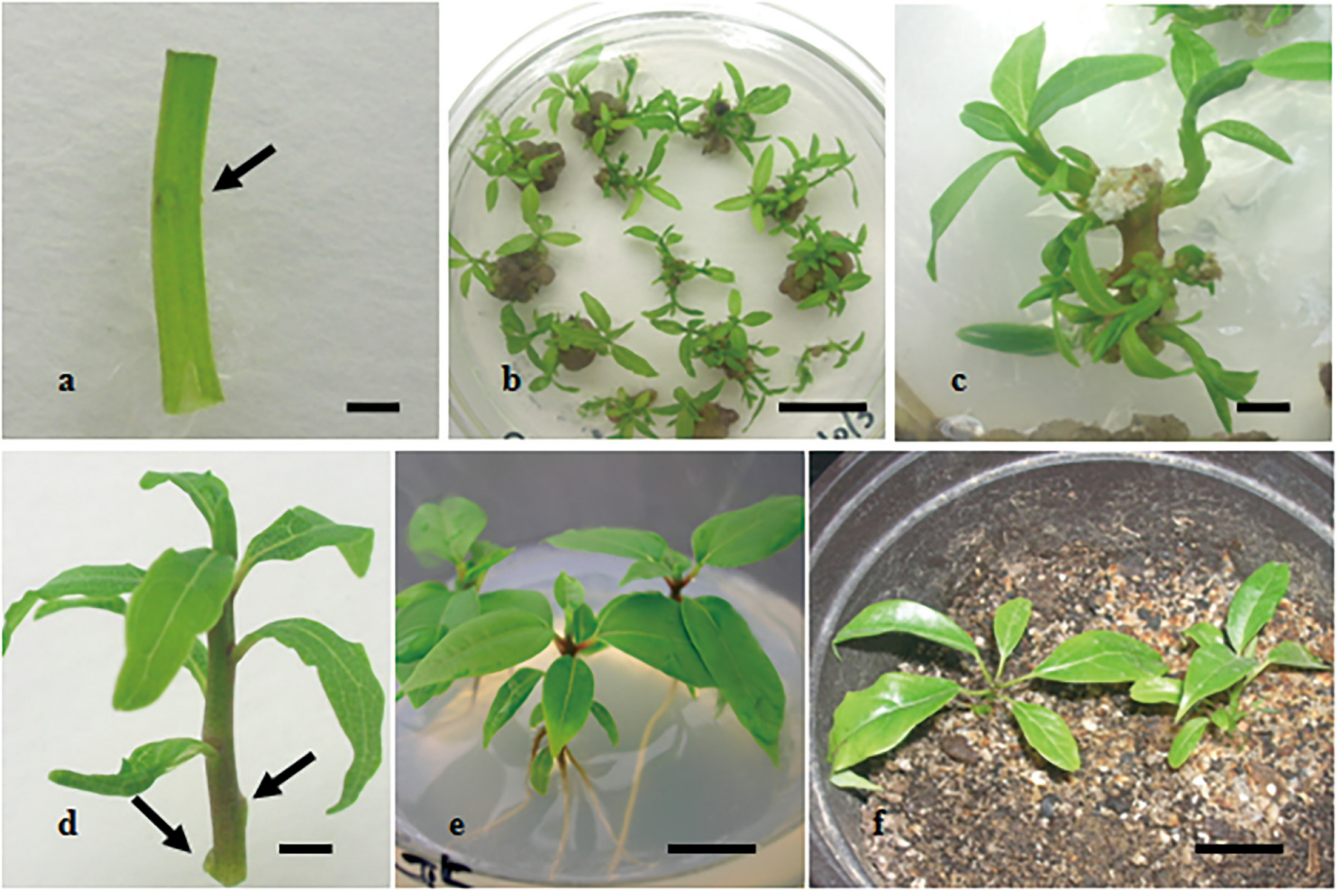
Plantlet regeneration from stem segment with nodule of embryo-cultured seedlings of *C*. *camphora*. a) a stem segment with one node (SN explant) for adventitious buds induction (arrow indicates the nodal region without axillary bud) bar: 0.1 cm; b) adventitious buds induced from SN explants, bar: 1.5 cm; c) direct organogenesis in SN explant, bar: 0.3 cm; d) a regenerated shoot with terminal bud from SN explants for elongation and root induction (arrow indicates excision points of lateral buds), bar: 0.3 cm; e) regenerated plantlet *in vitro*, bar: 1.0 cm; f) hardened plants, bar: 1.5 cm.

The SN explants (Fig 3A) were cultured on the adventitious bud induction medium, numerous adventitious buds could be observed developing directly from various positions of the explants (Fig 3B and 3C). Almost all induction media were able to directly induce adventitious buds from SN explants of mixed genetic background, with over 60% of induction frequency (Table 1). The highest adventitious bud induction frequency (83.3%), greatest number of buds per responding explant (4.1) and lowest callus induction frequency (23.3%) were obtained from S1 medium. In this research, after adventitious buds had been observed deriving from explants, a small amount of calli could be found on the explants on S1 medium. Other induction media resulted in calli excess growth. Additionally, quite a few calli could also be observed when explants subculture delayed.

**Table 1:** Effect of different media compositions on adventitious buds induction from SN explants of *C*. *camphora*.

Media	BA (mg/L)	2,4-D (mg/L)	IBA (mg/L)	CH (mg/L)	Frequency of adventitious buds induction (%)	No. of buds per responding explant	Frequency of callus induction (%)
**MS**	-	-	-	-	35.0^c^	1.3^d^	11.7^e^
**S 1**	2.0	0.5	-	-	83.3^a^	4.1^a^	23.3^d^
**S 2**	2.0	-	0.5	-	80.0^a^	4.1^a^	50.0^c^
**S 3**	3.0	0.5	-	-	65.0^b^	2.7^c^	73.3^a^ ^b^
**S 4**	3.0	-	0.5	-	85.0^a^	3.3^b^	80.0^a^
**S 5**	1.0	0.1	-	1000	61.7^b^	3.9^a^ ^b^	51.7^c^
**S 6**	1.0	-	0.1	1000	66.7^b^	3.8^a^ ^b^	63.3^b^ ^c^

**Note:** The data were collected after 2 weeks cultured on induction medium.

^a, b, c, d, e^Means in the same column not sharing a common superscript are significantly different (P<0.05).

The regeneration capability of the SN explants was compared for EC seedling lines EL1, EL2, EL3, EL4 and EL6. Although the induction frequencies for the different EC seedling lines were not significant, all frequencies were greater than 75% (Table 2). Different genotypes exhibited a statistically significant difference in the number of buds per responding explant (*P* < 0.05). The highest adventitious induction frequency (91.7%) and number of buds per responding explant (5.2) were obtained in EC seedling line EL6. For EL4, the adventitious induction frequency and number of buds per responding explant were 90.0% and 4.2, respectively. These two EC seedling lines could be considered as explants supplier for further gene transformation.

**Table 2:** Effect of explants donor genotype on adventitious buds induction from SN explants of *C*. *camphora*.

Genotype	Frequency of adventitious buds induction (%)	No. of buds per responding explant
**EL1**	81.7^a^	3.1^c^
**EL2**	83.3^a^	3.1^c^
**EL3**	78.3^a^	2.1^d^
**EL4**	90.0^a^	4.2^b^
**EL6**	91.7^a^	5.2^a^

**Note:** The data were collected after 2 weeks cultured on induction medium.

^a, b, c, d^Means in the same column not sharing a common superscript are significantly different (P<0.05).

In the adventitious bud induction experiments, the adventitious roots and buds both could be observed deriving from the SN explants, which were cultured on induction media without subculture in time (culture for 6~8 weeks). Therefore, there were omitting special rooting treatment in this research. Regenerated shoots from various induction media were transferred to hormone-free MS medium for elongation and rooting. After 2 weeks of adventitious bud induction on the different induction media (S1, S5 and S6 medium), the regenerated TS explants (>15 mm; Fig 3D) were excised from SN explants and transferred to MS medium. After 7 days, adventitious roots were observed from cultured shoots; as time progressed, elongation and rooting of shoots could be seen (Fig 3E). After 4 weeks in culture, the root induction frequency (68.3%) of shoots derived from medium S5 was significantly higher than the other two treatments. However, all the root induction frequencies were over 55% (Table 3). After culturing for another 4 weeks, frequencies increased to 80% (data not shown). In the rooting experiments, excess lateral buds could be observed developing multiple shoots, resulting in the regenerated shoot failing to elongate and root. Hence, excess lateral buds should be removed from TS explants (Fig 3D), to ensure no meristems found in the base of the shoot inserted into MS medium. Regenerated plants were successfully transferred to pots and showed normal morphological characteristics (Fig 3F). In this research, adventitious roots could be found from SN explants cultured on induction media; and multiple shoots could be obtained on hormone-free MS medium; these findings should indicate the material from EC seedlings of camphor tree could have higher capability in regenerating whole plantlet.

**Table 3:** Effect of different treatments on the root induction from regenerated shoot of *C*. *camphora*.

Treatment	Root induction frequency %	Multiple roots induction frequency %
**Regenerated shoots from S1**	58.3^b^	16.7^b^
**Regenerated shoots from S5**	68.3^a^	15.0^b^
**Regenerated shoots from S6**	56.7^b^	28.3^a^

**Note:** The data were collected after 4 weeks cultured on MS basal medium.

^a, b^Means in the same column not sharing a common superscript are significantly different (P<0.05).

Camphor tree is a woody species with a highly complex genetic background; therefore it is very difficult to regenerate plantlet *in vitro*. To date, there are very few reports describing high-frequency plantlet regeneration with respect to camphor tree. In 1998, Huang et al. developed a micropropagation protocol for camphor tree by using 3~5 mm shoot tips from newly emerged lateral buds [1]. However, this protocol was limited to clonal multiplication of camphor tree. Plantlet regeneration *via* somatic embryogenesis and cyclic secondary somatic embryogenesis of *C*. *camphora* was described by Du et al. 2006 [3] and Shi et al. 2010 [6]. The highest plantlet regeneration frequencies only were 33.3% and 54.25%, respectively. In the study of genetic transformation for camphor tree, transgenic embryogenic calli were obtained by *Agrobacterium*-mediated method. But these transgenic embryogenic calli could not successfully develop into somatic embryos and failed to regenerate plantlet [2, 5]. The researchers concluded that low regeneration frequencies, long regeneration periods and selective agents injury leaded to the failure of regeneration from transgenic embryogenic calli of camphor tree [2]. Till now, there have been few researches reported on successful transgenic plantlet regeneration *via* somatic embryogenesis of camphor tree. As a result, it was concluded that somatic embryogenesis could be an ineffective method for camphor tree transformation. In our current study, the regeneration frequency *via* direct organogenesis was significantly higher (up to 91.7%) than in previous reports. In consideration of sufficient explants supply, high-frequency regeneration and one-step regeneration induction, direct organogenesis of SN explants from the EC seedlings could become the more efficient regeneration method for the purposes of camphor tree transformation.

**Fig 4:**
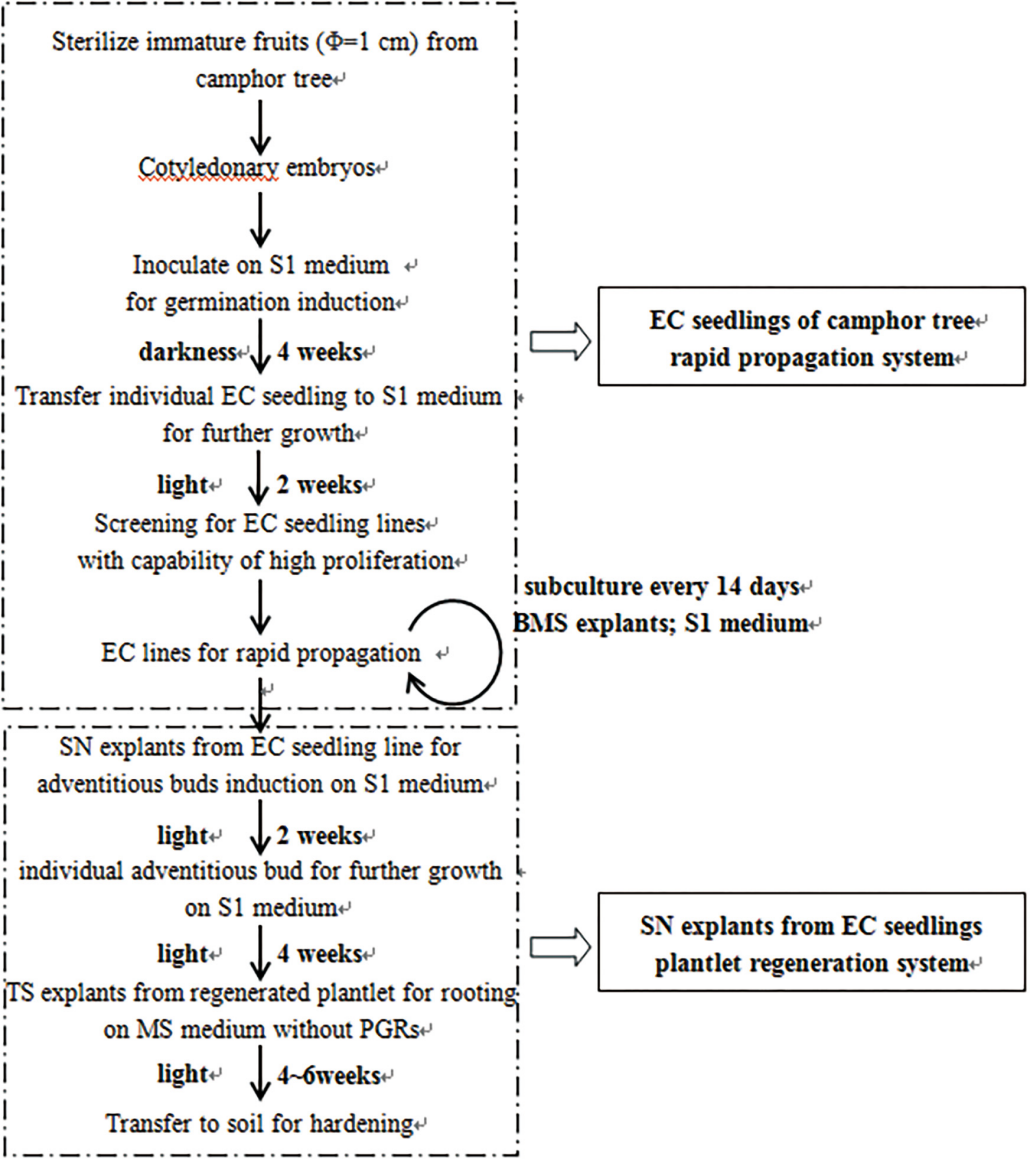
Schematic representation of the plantlet regeneration protocol for *C*. *camphora*.

In conclusion, this is the first report on plantlet regeneration *via* direct organogenesis by using nodal segments from embryo-cultured seedlings of camphor tree. An efficient and stable juvenile explants supplier was obtained by our research, and the juvenile material rapid propagation system and the plantlet regeneration system *via* organogenesis established are summarized in Fig 4. Our protocol described herein is efficient and reproducible. Propagation of EC seedlings and plantlet regeneration from SN explants of camphor trees include steps for embryo germination induction, screening and propagation of highly proliferation EC seedling lines, adventitious bud induction from SN explants, elongation and rooting induction of regenerated shoots. S1 medium was selected to use except root induction. Different explants from EC seedlings (SN, BMS and TS explants) were cultured on S1 or MS medium to achieve shoot regeneration, seedling propagation and regenerated shoot rooting. This protocol should allow the development of transformation protocol for camphor tree and have special reference to other woody plants with regeneration problems.
